# Stoma reversal after intended restorative rectal cancer resection in Denmark: nationwide population‐based study

**DOI:** 10.1002/bjs5.50340

**Published:** 2020-10-06

**Authors:** J. B. Jørgensen, R. Erichsen, B. G. Pedersen, S. Laurberg, L. H. Iversen

**Affiliations:** ^1^ Departments of Surgery Aarhus Denmark; ^2^ Clinical Epidemiology Aarhus Denmark; ^3^ Radiology Aarhus University Hospital Aarhus Denmark; ^4^ Department of Surgery Randers Regional Hospital Randers Denmark; ^5^ Danish Colorectal Cancer Group Copenhagen Denmark

## Abstract

**Background:**

Data on stoma reversal following restorative rectal resection (RRR) with a diverting stoma are conflicting. This study investigated a Danish population‐based cohort of patients undergoing RRR to evaluate factors predictive of stoma reversal during 3 years of follow‐up.

**Methods:**

Patients from national registries with rectal cancer undergoing RRR or Hartmann's procedure with curative intent between May 2001 and April 2012 were included. Patients with a diverting stoma were followed from the time of primary rectal cancer resection to date of stoma reversal, death, emigration, or end of 3‐year follow‐up. The cumulative incidence proportion (CIP) of stoma reversal at 1 and 3 years was calculated, treating death as a competing risk. Factors predictive of stoma reversal were explored using Cox regression analysis.

**Results:**

Of 6859 patients included, 35·7, 41·9 and 22·4 per cent respectively had a RRR with a diverting stoma, RRR without a stoma, and Hartmann's procedure with an end‐colostomy. In patients with a diverting stoma, the CIP of stoma reversal was 70·3 (95 per cent c.i. 68·4 to 72·1) per cent after 1 year, and 74·3 (72·5 to 76·0) per cent after 3 years. Neoadjuvant treatment (hazard ratio (HR) 0·75, 95 per cent c.i. 0·66 to 0·85), blood loss greater than 300 ml (HR 0·86, 0·76 to 0·97), anastomotic leak (HR 0·41, 0·33 to 0·50), T3 category (HR 0·63, 0·47 to 0·83), T4 category (HR 0·62, 0·42 to 0·90) and UICC stage IV (HR 0·57, 0·41 to 0·80) were possible predictors of delayed stoma reversal.

**Conclusion:**

In one‐quarter of the patients the diverting stoma had not been reversed 3 years after the intended RRR procedure.

## Graphical abstract

In this Danish population‐based cohort study, the stoma reversal rate during 3 years of follow‐up was estimated among patients undergoing intended restorative rectal cancer resection, and characteristics predictive of stoma reversal were explored. One‐quarter of patients had not had their diverting stoma reversed, and more than one‐half of patients with anastomotic leakage still had the stoma after 3 years.

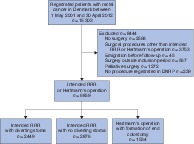

Stoma reversal after rectal surgery

## Abstract (es)

**Antecedentes:**

Los datos sobre el cierre del estoma (*stoma reversal*, SR) tras la exéresis el recto con intención reconstructiva (*restorative rectal resection*, RRR) y estoma derivativo (*diverting stoma*, DS) son contradictorios. Este estudio analizó los factores predictivos del SR en una cohorte danesa de base poblacional de pacientes sometidos a RRR con un seguimiento de 3 años.

**Métodos:**

Los pacientes con cáncer de recto a los que se realizó una RRR o una operación de Hartmann (*Hartmann's operation*, HO) con intención curativa desde mayo de 2001 hasta abril de 2012, se seleccionaron a partir de registros nacionales. Los pacientes con SD fueron seguidos desde la resección primaria del cáncer rectal hasta la fecha del SR, del fallecimiento, de su cambio de residencia o hasta el final del seguimiento (3 años). Se calculó la tasa de incidencia acumulada (*cumulative incidence proportion,* CIP) de RS a 1 y 3 años utilizando la muerte como factor de riesgo competitivo. Se identificaron los factores predictivos de SR mediante regresión múltiple de Cox.

**Resultados:**

De los 6.859 pacientes incluidos, el 35,7%, 41,9% y 22,4% tenían una RRR con DS, una RRR sin estoma y una HO con colostomía terminal, respectivamente. En pacientes con SD, el CIP de SR fue del 70,3% (i.c. del 95%: 68,4‐72,1) al año y del 74,3% (i.c. del 95%: 72,5‐76,0) a los 3 años. Se identificaron como posibles factores predictivos relacionados con el retraso del SR, el tratamiento neoadyuvante (cociente de riesgos instantáneos, *hazard ratio*, HR 0,75; i.c. del 95% 0,66‐0,85), una pérdida de sangre > 300 mL (HR 0,86; i.c. del 95% 0,76‐0,97), la fuga anastomótica (HR 0,41; i.c. del 95% 0,33‐0,50), las categorías T3 (HR 0,63; i.c. del 95% 0,47‐0,83) y T4 (HR 0,62; i.c. del 95% 0,42‐0,90) y el estadio IV UICC (HR 0,57; i.c. del 95%: 0,41‐0,80).

**Conclusión:**

En una cuarta parte de los pacientes no se había cerrado el estoma derivativo tres años después de la resección de cáncer rectal con intención reconstructiva.

## Introduction

In rectal cancer surgery, diverting stomas are created primarily to reduce the consequences of a possible anastomotic leak^1^, ^2^, ^3^, ^4^, ^5^, ^6^. Danish guidelines recommend a diverting stoma along with total mesorectal excision (TME) for the surgical treatment of mid and distal rectal cancer (tumour located 0–10 cm from the anal verge). A diverting stoma, however, is not created routinely as part of partial mesorectal excision (PME) for upper rectal cancer^7^.

Diverting stomas are usually reversed after 3 months, and possibly even earlier in selected patients with an uneventful postoperative course^8^. For patients in need of postoperative oncological treatment, however, stoma reversal can be postponed^8^.

Unfortunately, not all patients undergo reversion of the stoma after surgery. The risk of non‐reversal after rectal resection with an intended temporary diverting stoma varies between 3 and 32 per cent after 1·5–7·1 years^9^, ^10^, ^11^, ^12^, ^13^, ^14^, ^15^, ^16^, ^17^, ^18^, ^19^. Current literature^9^, ^10^, ^11^, ^12^, ^13^, ^14^, ^15^, ^16^, ^17^, ^18^, ^19^ suggests a median time to reversal of 1·5–5·1 years. The consequences of non‐reversal may be grave. Stoma‐related complications, ranging from minor inconvenience (such as leakage from the appliance and skin rash) to major disabilities (for instance, dehydration and electrolyte imbalance owing to a high stoma output, parastomal hernia and stoma prolapse), are common^6^, and may be associated with restriction in social activities and reduced quality of life^10^, ^15^, ^20^, ^21^.

**Fig. 1:**
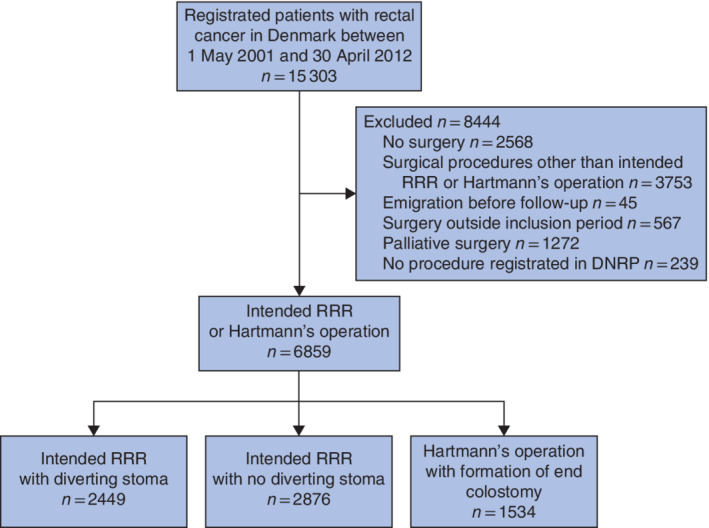
Flow diagram of 15 303 patients with first‐time rectal cancer diagnosed between 1 May 2001 and 30 April 2012, and treated at 21 hospitals in Denmark RRR, restorative rectal resection; DNRP, Danish National Registry of Patients.

Many studies have been conducted in a regional setting^11^, ^12^, ^13^, ^14^, ^15^, ^16^, ^17^, ^18^; therefore, a nationwide registry‐based cohort study of patients with rectal cancer, managed according to modern treatment regimens, may be important to have a clear picture of current clinical practice.

The primary aim of this study was to estimate the reversal rate for diverting stoma among patients undergoing rectal resection. Secondary aims included determining the characteristics of patients undergoing restorative rectal resection (RRR) and Hartmann's procedure, estimating the proportion of patients receiving a stoma, and exploring the predictors of diverting stoma reversal.

## Methods

This population‐based nationwide cohort study was approved by the National Board of Health (reference 3‐3013‐1272/1/), the Scientific Committee of the Danish Colorectal Cancer Group (DCCG) and the Danish Data Protection Agency (reference 2007‐58‐0010). The study was conducted in the setting of the entire Danish population^22^. The National Health Service in Denmark provides universal, tax‐supported healthcare to all citizens, guaranteeing free access to general practitioners and public hospitals^23^.

Data retrieved included clinical and demographic details, co‐morbidities, ASA grade, tumour characteristics (distance from anal verge), neoadjuvant treatment, pathological status, surgical procedures and approach, blood loss, postoperative anastomotic leakage and death within 3 years of the index surgical procedure.

### Data sources

Data were obtained from three registries: the DCCG database, Civil Registration System (CRS) and the Danish National Registry of Patients (DNRP).

Since May 2001, the DCCG database has been recording information on all patients with colorectal cancer with a completeness of 96–99 per cent^24^. The purpose of the database is to ensure uniform quality in the diagnosis and treatment of colorectal cancer in Denmark. All surgical departments across Denmark report prospectively on diagnostic staging, treatment and postoperative complications (occurring within 30 days of surgery)^24^. For this study, the DCCG database was used to identify the study cohort, as described below.

Since 1968, the Danish CRS has assigned a unique ten‐digit personal identification number (CPR number) to every resident in Denmark. The registry maintains information on date of birth, death, sex, residence and vital status. The civil registration number permits linkage within the healthcare system and among registries in Denmark^25^.

Finally, the DNRP has maintained records on all non‐psychiatric hospital admissions in Denmark since 1977. These include information on hospital diagnoses and procedures^26^. Data are collected for administrative purposes unrelated to research objectives. For instance, these data include the CPR number, dates of admission and discharge, and up to 20 discharge diagnoses, coded by physicians according to ICD‐10 from 1993 and onwards^27^. Since 1 January 1996, registration of surgical procedures in Denmark has been classified according to the Nordic Medico‐Statistical Committee (NOMESCO) classification of surgical procedures. DCCG and DNRP data were linked to obtain information on surgical events (stoma reversal) during follow‐up.

**Fig. 2:**
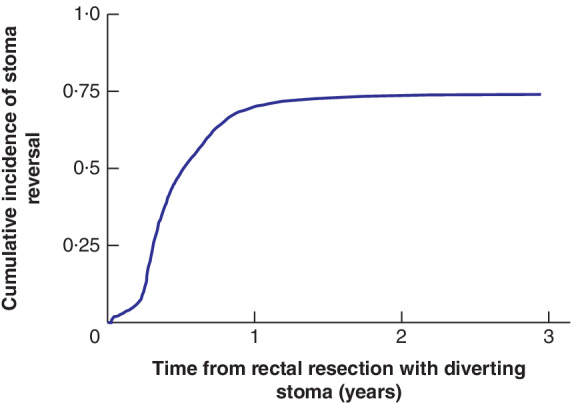
Cumulative incidence of stoma reversal during 3 years of follow‐up The cumulative incidence proportion was calculated with death treated as a competing risk.

### Study cohort

Patients diagnosed with rectal adenocarcinoma (located 15 cm or less from the anal verge), who underwent intended restorative rectal resection (TME, PME) or Hartmann's procedure with curative intent between 1 May 2001 and 30 April 2012, were identified from the DCCG database. Patients were excluded if they had surgical procedures other than intended RRR or Hartmann's operation, emigrated before the start of follow‐up, had palliative surgery, or had no registration of any surgical procedure in the DNRP despite registration in the DCCG database.

National guidelines^7^ recommend neoadjuvant long‐course (chemo)radiotherapy (CRT) (50 Gy in 25–28 fractions combined with 5‐fluorouracil) in patients with locally advanced rectal cancer. Alternatively, short‐course radiotherapy can be offered in some situations. Intended RRR and Hartmann's operation, performed as mesorectal excision, were done 8–10 weeks after completion of CRT. Short‐course radiotherapy with immediate surgery is not performed routinely in Denmark. All other patients underwent direct intended RRR or Hartmann's procedure. Selected patients with UICC stage II rectal cancer were offered 6 months of adjuvant chemotherapy, as were patients with UICC stage III disease who had not received neoadjuvant CRT^7^.

### Stoma reversal

Patients who underwent intended RRR with a diverting stoma were followed from the time of primary rectal cancer resection (index operation) to the date of stoma reversal (study endpoint), death, emigration or end of the 3‐year follow‐up. Information on stoma status (reversal or non‐reversal of the diverting stoma) during follow‐up was obtained via the DNRP, retrieving surgical procedure codes indicating stoma reversal according to the NOMESCO classification of surgical procedures.

The medical records of 9 per cent of patients who had a diverting stoma at the index operation were reviewed systemically to validate the stoma status as retrieved from the DNRP. Deviations from stoma status as retrieved from the DNRP were registered.

### Statistical analysis

Patients were characterized according to rectal resection procedure (diverting stoma, no stoma, and Hartmann's procedure), year of index operation and demographic details.

The cumulative incidence proportion (CIP) of stoma reversal at 1 and 3 years after rectal cancer surgery was calculated overall and for various patient‐ and disease‐related factors, treating death as a competing risk.

A Cox proportional hazards regression model was used to explore potential predictive factors for stoma reversal: period of surgery, age at operation, sex, BMI, Charlson Co‐morbidity Index (CCI) score, ASA grade, distance of primary tumour (lower edge) from anal verge (categorized as low (0–5 cm) *versus* mid/high (6–10/11–15 cm)), use of neoadjuvant CRT, surgical approach, intraoperative blood loss, anastomotic leakage, (y)pT category (according to T category) and UICC stage. Univariable and multivariable analyses were performed; multivariable analysis included all potential predictors for stoma reversal.

Stata® version 12.0 (StataCorp, College Station, Texas, USA) was used for statistical analysis.

## Results

Between 1 May 2001 and 30 April 2012, 15 303 patients with first‐time rectal cancer were treated at 21 hospitals in Denmark. Some 6859 of these patients underwent intended RRR or Hartmann's procedure with curative intent, and comprised the study cohort. Selection of the study cohort is detailed in *Fig*. *1*.

The proportion of patients undergoing intended RRR with a diverting stoma generally increased over the study period, from 29·7 to 42·4 per cent between 2001–2004 and 2009–2012 (*Table* *1*). In comparison, the proportion that had either RRR with no diverting stoma or Hartmann's procedure diminished gradually over the study interval.

**Table 1:** Characteristics of 6859 patients who had intended restorative rectal resection or Hartmann's operation in Denmark, 2001–2012

	Rectal resection with diverting stoma (*n* = 2449)	Rectal resection with no stoma (*n* = 2876)	Hartmann operation (*n* = 1534)
**Period of surgery**			
May 2001 to December 2004	677 (29·7)	979 (43·0)	620 (27·2)
January 2005 to December 2008	865 (35·4)	1090 (44·6)	490 (20·0)
January 2009 to April 2012	907 (42·4)	807 (37·7)	424 (19·8)
**Age (years)** *	65 (20–91)	67 (29–97)	75 (28–94)
**Sex**			
M	1551 (63·3)	1571 (54·6)	943 (61·5)
F	898 (36·7)	1305 (45·4)	591 (38·5)
**BMI (kg/m^2^)**			
0–19	101 (4·1)	155 (5·4)	109 (7·1)
20–24	853 (34·8)	1017 (35·4)	447 (29·1)
25–29	781 (31·9)	878 (30·5)	339 (22·1)
≥ 30	277 (11·3)	296 (10·3)	157 (10·2)
Missing	437 (17·8)	530 (18·4)	482 (31·4)
**Charlson Co‐morbidity Index score**			
0	2079 (84·9)	2390 (83·1)	1082 (70·5)
1–2	327 (13·4)	424 (14·7)	363 (23·7)
≥ 3	43 (1·8)	62 (2·2)	89 (5·8)
**ASA grade**			
I	745 (30·4)	903 (31·4)	178 (11·6)
II	1402 (57·2)	1567 (54·5)	789 (51·4)
III	250 (10·2)	332 (11·5)	466 (30·4)
IV	5 (0·2)	22 (0·8)	52 (3·4)
Missing	47 (1·9)	52 (1·8)	49 (3·2)
**Distance of primary tumour (lower edge) from anal verge (cm)**			
0–5	207 (8·5)	87 (3·0)	173 (11·3)
6–10	1515 (61·9)	824 (28·7)	823 (53·7)
11–15	704 (28·7)	1924 (66·9)	495 (32·3)
Missing	23 (0·9)	41 (1·4)	43 (2·8)
**Neoadjuvant (chemo)radiotherapy**	719 (29·4)	274 (9·5)	373 (24·3)
**Surgical approach**			
Laparotomy†	1887 (77·1)	2133 (74·2)	1277 (83·2)
Laparoscopy	562 (22·9)	743 (25·8)	256 (16·7)
Missing	0 (0)	0 (0)	1 (0·1)
**Intraoperative blood loss (ml)**			
0–300	1212 (49·5)	1364 (47·4)	536 (34·9)
> 300	1207 (49·3)	1443 (50·2)	943 (61·5)
Missing	30 (1·2)	69 (2·4)	55 (3·6)
**Anastomotic leak**	286 (11·7)	373 (13·0)	n.a.
**(y)pT category**			
T1	203 (8·3)	187 (6·5)	46 (3·0)
T2	528 (21·6)	467 (16·2)	259 (16·9)
T3	1316 (53·7)	1571 (54·6)	734 (47·9)
T4	97 (4·0)	193 (6·7)	208 (13·6)
Missing	305 (12·5)	458 (15·9)	287 (18·7)
**UICC stage**			
I	655 (26·7)	613 (21·3)	283 (18·4)
II	763 (31·2)	1013 (35·2)	526 (34·3)
III	839 (34·3)	943 (32·8)	430 (28·0)
IV‡	175 (7·1)	277 (9·6)	270 (17·6)
Missing	17 (0·7)	30 (1·0)	25 (1·6)
**Death within 3 years of surgery**	394 (16·1)	551 (19·2)	661 (43·1)

Values in parentheses are percentages unless indicated otherwise;

* values are median (range).

† Includes intended laparoscopic surgery but converted to open surgery.

‡ Patients with UICC stage IV rectal cancer had treatment of distant metastasis with curative intent together with intended restorative rectal resection or Hartmann's operation during either the index procedure or an independent procedure done close to the index procedure. n.a., Not applicable.

Patients who had RRR with formation of a diverting stoma and patients without a stoma were comparable with respect to age, BMI, co‐morbidity, T category, UICC stage, surgical approach and anastomotic leak rate (*Table* *1*). However, male sex, tumour location in the lower or mid rectum, use of neoadjuvant CRT and higher blood loss were more common in patients with a diverting stoma than in those without. Demographics, tumour characteristics and treatment regimens are summarized in *Table* *1*.

A total of 225 medical records were reviewed to validate stoma status at 3‐year follow‐up. Discrepancies were found for seven patients (3·1 (95 per cent c.i. 0·8 to 5·4) per cent) in comparison with their register‐based status. Discrepancies were due to incorrect NOMESCO registration of stoma reversal during follow‐up (both missing registration of reversal and registration of reversal never mentioned in medical records) or incorrect registration of the CPR number.

### Cumulative incidence proportion of stoma reversal

The CIP of diverting stoma reversal was 0·70 (95 per cent c.i. 0·68 to 0·72) after 1 year and 0·74 (0·73 to 0·76) after 3 years (*Fig*. *2* and *Table* *2*). Median time to stoma reversal was 173·0 (range 1–1075) days.

**Table 2:** Cumulative incidence proportions at 1 and 3 years for stoma reversal in patients who had intended restorative rectal cancer resection with diverting stoma in Denmark, 2001–2012

		Cumulative incidence proportion†, ‡	
	Patients with rectal resection and diverting stoma*	1 year	3 years
**Overall**	2449	0·70 (0·68, 0·72)	0·74 (0·73, 0·76)
**Period of surgery**			
May 2001 to December 2004	677 (27·6)	0·68 (0·65, 0·72)	0·71 (0·67, 0·74)
January 2005 to December 2008	865 (35·3)	0·69 (0·66, 0·72)	0·74 (0·70, 0·76)
January 2009 to April 2012	907 (37·0)	0·73 (0·70, 0·76)	0·78 (0·75, 0·80)
**Age (years)**			
≤ 65	1240 (50·6)	0·73 (0·70, 0·75)	0·77 (0·74, 0·79)
> 65	1209 (49·4)	0·68 (0·65, 0·71)	0·72 (0·69, 0·74)
**Sex**			
M	1551 (63·3)	0·72 (0·69, 0·75)	0·75 (0·72, 0·78)
F	898 (36·7)	0·69 (0·67, 0·72)	0·74 (0·71, 0·76)
**BMI (kg/m** ^**2**^ **)**			
0–19	101 (4·1)	0·64 (0·54, 0·73)	0·65 (0·55, 0·74)
20–24	853 (34·8)	0·72 (0·68, 0·75)	0·75 (0·72, 0·78)
25–29	781 (31·9)	0·74 (0·71, 0·77)	0·78 (0·75, 0·81)
≥ 30	277 (11·3)	0·70 (0·64, 0·75)	0·73 (0·68, 0·78)
Missing	437 (17·8)	–	–
**Charlson Co‐morbidity Index score**			
0	2079 (84·9)	0·71 (0·69, 0·73)	0·75 (0·73, 0·77)
1–2	327 (13·4)	0·66 (0·61, 0·71)	0·70 (0·64, 0·73)
≥ 3	43 (1·8)	0·58 (0·42, 0·71)	0·60 (0·44, 0·73)
**ASA grade**			
I	745 (30·4)	0·75 (0·71, 0·77)	0·78 (0·75, 0·81)
II	1402 (57·2)	0·70 (0·67, 0·72)	0·74 (0·71, 0·76)
III–IV	255 (10·4)	0·60 (0·54, 0·66)	0·64 (0·58, 0·69)
Missing	47 (1·9)	–	–
**Distance of primary tumour (lower edge) from anal verge (cm)** **§**			
0–5	207 (8·5)	0·63 (0·56, 0·69)	0·68 (0·61, 0·74)
6–10	1 515 (61·9)	0·70 (0·68, 0·73)	0·74 (0·72, 0·76)
11–15	704 (28·7)	0·72 (0·69, 0·75)	0·76 (0·73, 0·79)
Missing	23 (0·9)	–	–
**Neoadjuvant (chemo)radiotherapy**			
No	1730 (70·6)	0·73 (0·71, 0·75)	0·77 (0·75, 0·79)
Yes	719 (29·4)	0·64 (0·60, 0·67)	0·68 (0·64, 0·71)
**Surgical approach**			
Laparotomy	1887 (77·1)	0·71 (0·67, 0·73)	0·76 (0·72, 0·79)
Laparoscopy	562 (22·9)	0·70 (0·68, 0·72)	0·74 (0·72, 0·76)
**Intraoperative blood loss (ml)**			
0–300	1212 (49·5)	0·75 (0·72, 0·77)	0·79 (0·76, 0·81)
> 300	1207 (49·3)	0·66 (0·63, 0·69)	0·70 (0·67, 0·73)
Missing	30 (1·2)	–	–
**Anastomotic leak**			
No	2162 (88·3)	0·75 (0·73, 0·77)	0·78 (0·76, 0·80)
Yes	286 (11·7)	0·33 (0·29, 0·38)	0·45 (0·39, 0·51)
Missing	1 (0·0)	–	–
**(y)pT category**			
T1	203 (8·3)	0·80 (0·74, 0·85)	0·83 (0·77, 0·88)
T2	528 (21·6)	0·75 (0·71, 0·78)	0·79 (0·75, 0·82)
T3	1316 (53·7)	0·68 (0·65, 0·71)	0·73 (0·70, 0·75)
T4	97 (4·0)	0·61 (0·50, 0·70)	0·66 (0·56, 0·74)
Missing	305 (12·5)	–	–
**UICC stage**			
I	655 (26·7)	0·77 (0·74, 0·80)	0·80 (0·77, 0·83)
II	763 (31·2)	0·74 (0·71, 0·77)	0·77 (0·73, 0·79)
III	839 (34·3)	0·66 (0·63, 0·69)	0·71 (0·68, 0·74)
IV	175 (7·1)	0·50 (0·42, 0·57)	0·57 (0·50, 0·64)
Missing	17 (0·7)	–	–

Values in parentheses are

* percentages and

† 95 per cent confidence intervals.

‡ Calculated treating death as a competing risk.

§ Measured by rigid proctoscopy.

Patients with an ASA grade above II, neoadjuvant CRT, blood loss greater than 300 ml, anastomotic leakage, T category above T2 and UICC stage greater than II had a particularly low CIP of stoma reversal at 1 year after the index operation (*Table* *2*). Similarly, at 3 years after the index operation, patients aged above 65 years (CIP 0·72, 95 per cent c.i. 0·69 to 0·74), with ASA grade above II (CIP 0·64, 0·58 to 0·69), neoadjuvant CRT (CIP 0·68, 0·64 to 0·71), blood loss greater than 300 ml (CIP 0·70, 0·67 to 0·73), anastomotic leakage (CIP 0·45, 0·39 to 0·51), T3 category (CIP 0·73, 0·70 to 0·75) or T4 (CIP 0·66, 0·56 to 0·74), and UICC stage III (CIP 0·71, 0·68 to 0·74) or IV (CIP 0·57, 0·50 to 0·64) had particularly low CIP values (*Table* *2*).

Patients with UICC stage I disease had the highest CIP of stoma reversal at 3 years after the index operation (0·80). Patients with anastomotic leakage had the lowest CIP of stoma reversal at 3 years after the index operation (0·45).

### Factors predictive of delay in stoma reversal

Higher ASA grade (II and III–IV), neoadjuvant CRT, blood loss greater than 300 ml, anastomotic leakage, advanced T category (T2, T3 and T4) and more advanced UICC stage (II, III and IV) were associated with a risk of delay in time to stoma reversal in univariable analysis (*Table* *3*).

In multivariable analysis, neoadjuvant CRT (hazard ratio (HR) 0·75, 95 per cent c.i. 0·66 to 0·85), blood loss greater than 300 ml (HR 0·86, 0·76 to 0·97), anastomotic leakage (HR 0·41, 0·33 to 0·50), T3 category (HR 0·63, 0·47 to 0·83), T4 category (HR 0·62, 0·42 to 0·90) and UICC stage IV (HR 0·57, 0·41 to 0·80) were found to be independent predictors of delay in time to stoma reversal (*Table* *3*).

**Table 3:** Crude and adjusted hazard ratios (0–3 years) to determine factors predictive of delay in stoma reversal in patients who had intended restorative rectal cancer resection with diverting stoma in Denmark, 2001–2012

		Hazard ratio*	
	No. of subjects (n = 2449)	Crude†	Adjusted‡
**Period of surgery**			
May 2001 to December 2004	677 (27·6)	1·00 (reference)	1·00 (reference)
January 2005 to December 2008	865 (35·3)	0·96 (0·85, 1·08)	1·08 (0·91, 1·28)
January 2009 to April 2012	907 (37·0)	1·01 (0·90, 1·13)	1·07 (0·90, 1·28)
**Age (years)**			
≤ 65	1240 (50·6)	1·00 (reference)	1·00 (reference)
> 65	1209 (49·4)	0·95 (0·87, 1·04)	0·92 (0·82, 1·03)
**Sex**			
M	1551 (63·3)	0·98 (0·89, 1·08)	1·05 (0·94, 1·19)
F	898 (36·7)	1·00 (reference)	1·00 (reference)
**BMI (kg/m^2^)**			
0–19	101 (4·1)	1·00 (reference)	1·00 (reference)
20–24	853 (34·8)	1·13 (0·87, 1·45)	1·12 (0·84, 1·49)
25–29	781 (31·9)	1·17 (0·91, 1·51)	1·17 (0·87, 1·56)
≥ 30	277 (11·3)	1·06 (0·80, 1·39)	1·08 (0·79, 1·48)
Missing	437 (17·8)	–	–
**Charlson Co‐morbidity Index score**			
0	2079 (84·9)	1·00 (reference)	1·00 (reference)
1–2	327 (13·4)	0·93 (0·81, 1·07)	0·99 (0·84, 1·17)
≥ 3	43 (1·8)	0·82 (0·56, 1·21)	0·77 (0·49, 1·20)
**ASA grade**			
I	745 (30·4)	1·00 (reference)	1·00 (reference)
II	1402 (57·2)	0·87 (0·79, 0·96)	0·91 (0·80, 1·03)
III–IV	255 (10·4)	0·75 (0·63, 0·89)	0·82 (0·65, 1·02)
Missing	47 (1·9)	–	–
**Distance of primary tumour (lower edge) from anal verge (cm)**			
0–5	207 (8·5)	0·84 (0·69, 1·01)	0·84 (0·66, 1·06)
6–10	1515 (61·9)	1·03 (0·93, 1·14)	1·11 (0·97, 1·26)
11–15	704 (28·7)	1·00 (reference)	1·00 (reference)
Missing	23 (0·9)	–	–
**Neoadjuvant (chemo)radiotherapy**			
No	719 (29·4)	0·76 (0·69, 0·85)	0·75 (0·66, 0·85)
Yes	1730 (70·6)	1·00 (reference)	1·00 (reference)
**Surgical approach**			
Laparoscopy	562 (22·9)	1·00 (reference)	1·00 (reference)
Laparotomy	1887 (77·1)	0·96 (0·86, 1·07)	1·00 (0·87, 1·16)
**Intraoperative blood loss (ml)**			
0–300	1212 (49·5)	1·00 (reference)	1·00 (reference)
> 300	1207 (49·3)	0·80 (0·73, 0·88)	0·86 (0·76, 0·97)
Missing	30 (1·2)	–	–
**Anastomotic leak**			
Yes	286 (11·7)	0·42 (0·35, 0·50)	0·41 (0·33, 0·50)
No	2162 (88·3)	1·00 (reference)	1·00 (reference)
Missing	1 (0·0)	–	–
**(y)pT category**			
T1	203 (8·3)	1·00 (reference)	1·00 (reference)
T2	528 (21·6)	0·83 (0·69, 0·99)	0·86 (0·70, 1·05)
T3	1316 (53·7)	0·63 (0·53, 0·74)	0·63 (0·47, 0·83)
T4	97 (4·0)	0·51 (0·39, 0·69)	0·62 (0·42, 0·90)
Missing	305 (12·5)	–	–
**UICC stage**			
I	655 (26·7)	1·00 (reference)	1·00 (reference)
II	763 (31·2)	0·86 (0·76, 0·97)	1·23 (0·94, 1·63)
III	839 (34·3)	0·65 (0·58, 0·73)	0·83 (0·65, 1·05)
IV	175 (7·1)	0·42 (0·34, 0·52)	0·57 (0·41, 0·80)
Missing	17 (0·7)	–	–

Values in parentheses are percentages unless otherwise stated;

* values in parentheses are 95 per cent confidence intervals.

† Cox regression analysis; a hazard ratio below 1 indicates a reduced ‘risk’ of stoma reversal (early stoma closure).

‡ Mutually adjusted.

## Discussion

The main finding of this nationwide population‐based cohort study was the fact that an unexpectedly low rate of only 74·3 per cent of patients with rectal cancer and an intended temporary diverting stoma had their stoma reversed during 3 years of follow‐up. As more than one‐third of patients having intended RRR had a diverting stoma, a substantial number of patients end up with a permanent stoma.

Previous studies^13^, ^19^ investigating stoma reversal rates after anterior resection have shown great variation in permanent stoma rates. The majority of these studies were based on smaller patient cohorts (ranging from 50 to 523^11^, ^12^, ^13^, ^14^, ^16^, ^18^), originating from single centres or a local region.

Multicentre studies including only patients with rectal cancer^9^, ^10^, ^15^, ^17^ reported a 17–25 per cent^9^, ^10^ risk of a permanent stoma after intended rectal resection together with a diverting stoma, similar to findings in the present study.

In this study, the independent predictive factors for stoma non‐reversal within 3 years were anastomotic leakage, advanced UICC stage IV, T3 and T4 category, use of neoadjuvant CRT and perioperative blood loss greater than 300 ml. These results are in line with previous studies, which showed that increasing age, co‐morbidity^10^, ^12^, ^17^, ^18^, ^20^, locally advanced or metastatic disease^11^, ^12^, ^16^, ^17^, any postoperative complication^11^, ^12^, ^15^, ^17^ and anastomotic leakage^14^, ^16^, ^18^ increased the probability of a permanent stoma after anterior resection.

A retrospective multicentre English study^10^ (2001–2003) of 6582 patients (964 with a diverting stoma) examined the use of loop ileostomy after low anterior resection, and found that increasing age and co‐morbidity increased the probability of a permanent stoma. However, only a relatively small proportion of patients (14·6 per cent) had a diverting stoma at time of primary surgery, and none of the patients had neoadjuvant CRT. In the present cohort, neoadjuvant CRT was a significant predictor of stoma non‐reversal. Differences in the baseline characteristics of the patient cohorts in the two studies may have led to non‐comparable results.

A Swedish retrospective multicentre study^9^ including 3564 patients undergoing rectal cancer surgery observed that higher level of education increased the probability of early stoma reversal. Postoperative complications, adjuvant chemotherapy, advanced UICC stage and advanced ASA grade were associated with delay in time to stoma reversal. This study differed from the present study, as it provided no information on the proportion of patients who received a stoma at the index operation. In addition, follow‐up was short (total of 1·5 years) and there was no validation of clinical information from Swedish registries.

Two prospective multicentre studies^15^, ^17^ examined risk factors for permanent stoma following TME with formation of either a diverting colostomy (37 per cent^15^ and 3 per cent^17^) or ileostomy (63 per cent^15^ and 97 per cent^17^). Co‐morbidity, metastatic disease, anastomotic leakage, deteriorated anorectal function, postoperative complications of any kind, and secondarily constructed stomas were significant risk factors. However, results from these studies^15^, ^17^ cannot be extrapolated directly, as the risk of a permanent stoma after construction of a diverting colostomy may be different from that for a diverting ileostomy.

Interestingly, the present study shows that an increasing proportion of patients underwent RRR as time passed. Whereas 29·7 per cent had restorative surgery in 2001–2004, the proportion was 42·4 per cent in 2009–2012. This general change in surgical approach may partly result from the revision of national guidelines in 2009 and methodological alignment between departments.

Changes in surgical approach over time with extended use of diverting stomas have not been found in other studies, and suggest that surgeons may be increasingly cautious following RRR. However, recent studies from the Netherlands^28^, ^29^ found no difference in short‐term postoperative complication rates between patients undergoing rectal cancer resection with a diverting stoma routinely and those in whom a diverting stoma was created only in highly selected patients. Equally, the findings from the present study suggest that long‐term complications might be another important issue that requires attention in the preoperative setting. Selecting specific patient groups for stoma construction was found to be a key element in optimizing patient outcomes. These findings may change the tendency towards a more selective approach to construction of diverting stoma in future.

The strengths of this study are the nationwide prospective design, the large patient cohort, and the long follow‐up of 3 years. Data were retrieved from three highly reliable registries, with prospective sampling of data and high data completeness^25^, ^26^, ^30^. Unlike most previous studies, this study was based on data from the current time period, where treatment regimens comply with modern standards.

Although data were retrieved from validated registries, the validity and methods of data reporting might differ between hospitals. This is one of the limitations of the study, as coding errors related to operative procedures may occur. However, discrepancies between medical records and databases regarding stoma status at the 3‐year follow‐up were low in the sample survey of 9·2 per cent of patients who had rectal resection with formation of a diverting stoma.

This study shows that one‐quarter of patients had not undergone stoma reversal 3 years after restorative rectal cancer resection in Denmark. This proportion was doubled where postoperative management was complicated by an anastomotic leak.

## References

[1] Matthiessen P , Hallböök O , Rutegård J , Simert G , Sjödahl R. Defunctioning stoma reduces symptomatic anastomotic leakage after low anterior resection of the rectum for cancer: a randomized multicenter trial. Ann Surg 2007; 246: 207–214. 17667498 10.1097/SLA.0b013e3180603024 PMC1933561

[2] Gastinger I , Marusch F , Steinert R , Wolff S , Koeckerling F , Lippert H. Protective defunctioning stoma in low anterior resection for rectal carcinoma. Br J Surg 2005; 92: 1137–1142. 15997447 10.1002/bjs.5045

[3] Karanjia ND , Corder AP , Holdsworth PE , Heald RJ . Risk of peritonitis and fatal septicaemia and the need to defunction the low anastomosis. Br J Surg 1991; 78: 196–198. 2015471 10.1002/bjs.1800780221

[4] Marusch F , Koch A , Schmidt U , Geibetaler S , Dralle H , Saeger HD *et al* Value of a protective stoma in low anterior resections for rectal cancer. Dis Colon Rectum 2002; 45: 1164–1171. 12352230 10.1007/s10350-004-6384-9

[5] Dehni N , Schlegel RD , Cunningham C , Guiguet M , Tiret E , Parc R . Influence of a defunctioning stoma on leakage rates after low colorectal anastomosis and colonic J pouch–anal anastomosis. Br J Surg 1998; 85: 1114–1117. 9718009 10.1046/j.1365-2168.1998.00790.x

[6] Chow A , Tilney HS , Paraskeva P , Jeyarajah S , Zacharakis E , Purkayastha S . The morbidity surrounding reversal of defunctioning ileostomies: a systematic review of 48 studies including 6107 cases. Int J Colorectal Dis 2009; 24: 711–723. 19221766 10.1007/s00384-009-0660-z

[7] Danish Colorectal Cancer Group . [*Guidelines for the Diagnosis and Management of Colorectal Cancer*]; 2009 https://dccg.dk/wp‐content/uploads/2017/08/Retningslinier2009revOKT2010.pdf [accessed 1 April 2020].

[8] Alves A , Panis Y , Lelong B , Dousset B , Benoist S , Vicaut E. Randomized clinical trial of early *versus* delayed temporary stoma closure after proctectomy. Br J Surg 2008; 95: 693–698. 18446781 10.1002/bjs.6212

[9] Gustafsson CP , Gunnarsson U , Dahlstrand U , Lindforss U . Loop‐ileostomy reversal – patient‐related characteristics influencing time to closure. Int J Colorectal Dis 2018; 33: 593–600. 29508050 10.1007/s00384-018-2994-x PMC5899111

[10] David GG , Slavin JP , Willmott S , Corless DJ , Khan AU , Selvasekar CR . Loop ileostomy following anterior resection: is it really temporary? Colorectal Dis 2010; 12: 428–432. 19226365 10.1111/j.1463-1318.2009.01815.x

[11] Gessler B , Haglind E , Angenete E . Loop ileostomies in colorectal cancer patients – morbidity and risk factors for nonreversal. J Surg Res 2012; 178: 708–714. 22940030 10.1016/j.jss.2012.08.018

[12] Pan HD , Peng YF , Wang L , Li M , Yao YF , Zhao J *et al* Risk factors for nonclosure of a temporary defunctioning ileostomy following anterior resection of rectal cancer. Dis Colon Rectum 2016; 59: 94–100. 26734966 10.1097/DCR.0000000000000520

[13] Lordan JT , Heywood R , Shirol S , Edwards DP . Following anterior resection for rectal cancer, defunctioning ileostomy closure may be significantly delayed by adjuvant chemotherapy: a retrospective study. Colorectal Dis 2007; 9: 420–422. 17504338 10.1111/j.1463-1318.2006.01178.x

[14] Dinnewitzer A , Jäger T , Nawara C , Buchner S , Wolfgang H , Öfner D . Cumulative incidence of permanent stoma after sphincter preserving low anterior resection of mid and low rectal cancer. Dis Colon Rectum 2013; 56: 1134–1142. 24022530 10.1097/DCR.0b013e31829ef472

[15] den Dulk M , Smit M , Peeters KC , Kranenbarg EM , Rutten HJ , Wiggers T *et al* A multivariate analysis of limiting factors for stoma reversal in patients with rectal cancer entered into the total mesorectal excision (TME) trial: a retrospective study. Lancet Oncol 2007; 8: 297–303. 17395102 10.1016/S1470-2045(07)70047-5

[16] Bailey CMH , Wheeler JM , Birks M , Farouk R . The incidence and causes of permanent stoma after anterior resection. Colorectal Dis 2003; 5: 331–334. 12814411 10.1046/j.1463-1318.4.s1.1_78.x

[17] Lindgren R , Hallböök O , Rutegård J , Sjödahl R , Matthiessen P . What is the risk for a permanent stoma after low anterior resection of the rectum for cancer? A six‐year follow‐up of a multicenter trial. Dis Colon Rectum 2011; 54: 41–47. 21160312 10.1007/DCR.0b013e3181fd2948

[18] Chiu A , Chan HT , Brown CJ , Raval MJ , Phang PT . Failing to reverse a diverting stoma after lower anterior resection of rectal cancer. Am J Surg 2014; 207: 708–711. 24791631 10.1016/j.amjsurg.2013.12.016

[19] Maggiori L , Bretagnol F , Lefevre JH , Ferron M , Vicaut E , Panis Y. Conservative management is associated with a decreased risk of definitive stoma after anastomotic leakage complicating sphincter‐saving resection for rectal cancer. Colorectal Dis 2011; 13: 632–637. 20236150 10.1111/j.1463-1318.2010.02252.x

[20] Kairaluoma M , Rissanen H , Kultti V , Mecklin JP , Kellokumpu I. Outcome of temporary stomas. Dig Surg 2002; 19: 45–51. 11961355 10.1159/000052005

[21] O'Leary DP , Fide CJ , Foy C , Lucarotti ME . Quality of life after low anterior resection with total mesorectal excision and temporary loop ileostomy for rectal carcinoma. Br J Surg 2001; 88: 1216–1220. 11531870 10.1046/j.0007-1323.2001.01862.x

[22] Frank L. When an entire country is a cohort. Science 2000; 287: 2398–2399. 10766613 10.1126/science.287.5462.2398

[23] Schmidt M , Schmidt SA , Adelborg K , Sundbøll J , Laugesen K , Ehrenstein V *et al* The Danish health care system and epidemiological research: from health care contacts to database records. Clin Epidemiol 2019; 11: 563–591. 31372058 10.2147/CLEP.S179083 PMC6634267

[24] Ingeholm P , Gogenur I , Iversen LH . Danish Colorectal Cancer Group database. Clin Epidemiol 2016; 8: 465–468. 27822086 10.2147/CLEP.S99481 PMC5094575

[25] Schmidt M , Pedersen L , Sorensen HT . The Danish Civil Registration System as a tool in epidemiology. Eur J Epidemiol 2014; 29: 541–549. 24965263 10.1007/s10654-014-9930-3

[26] Schmidt M , Schmidt SA , Sandegaard JL , Ehrenstein V , Pedersen L , Sørensen HT . The Danish National Patient Registry: a review of content, data quality, and research potential. Clin Epidemiol 2015; 7: 449–490. 26604824 10.2147/CLEP.S91125 PMC4655913

[27] Lynge E , Sandegaard JL , Rebolj M . The Danish national patient register. Scand J Public Health 2011; 39: 30–33. 21775347 10.1177/1403494811401482

[28] Snijders HS , van Leersum NJ , Henneman D , de Vries AC , Tollenaar RA , Stiggelbout AM *et al* Optimal treatment strategy in rectal cancer surgery: should we be cowboys or chickens? Ann Surg Oncol 2015; 22: 3582–3589. 25691277 10.1245/s10434-015-4385-7 PMC4565862

[29] Blok RD , Stam R , Westerduin E , Borstlap WA , Hompes R , Bemelman WA *et al* Impact of an institutional change from routine to highly selective diversion of a low anastomosis after TME for rectal cancer. Eur J Surg Oncol 2018; 44: 1220–1225. 29685761 10.1016/j.ejso.2018.03.033

[30] Bulow, S , Harling H , Iversen LH , Ladelund S ; Danish Colorectal Cancer Group. Improved survival after rectal cancer in Denmark. Colorectal Dis 2010; 12: e37–e42. 19614669 10.1111/j.1463-1318.2009.02012.x

